# Supplementary material for: Enormous-stiffness-changing polymer networks by glass transition mediated microphase separation
Source: Nat Commun. 2022 Nov 10;13:6821. doi: 10.1038/s41467-022-34677-9 (PMC9649666; doi:10.1038/s41467-022-34677-9)
Supplement: Supplementary file 1 — Supplementary Information [file 41467_2022_34677_MOESM1_ESM.pdf]

Supplementary Information

Enormous-stiffness-changing polymer networks by glass transition mediated microphase separation

Lie Chen[1,2], Cong Zhao[1], Jin Huang[1], Jiajia Zhou[1]*, Mingjie Liu[1,3]*

Correspondence to: liumj@buaa.edu.cn and jjzhou@buaa.edu.cn

1. Key Laboratory of Bio-Inspired Smart Interfacial Science and Technology of Ministry of Education, School of Chemistry, Beihang University, Beijing, 100191, P. R. China.
2. Nerve-Machine Integration and Cognitive Competition Center, Beijing Machine and Equipment institute, Beijing 100854, P. R. China.
3. International Research Institute for Multidisciplinary Science, Beihang University, Beijing 100191, China.

Table of Contents

1. **Materials**
2. **Instrumentation**
3. **Supplementary Figures (1-16)**
4. **Supplementary Tables (1-3)**
5. **References**

1. Materials

All of the following chemical reagents were used as received from Sigma-Aldrich. [N-Isopropyl acrylamide (NIPAm), Ethyleneglycol dimethacrylate (EGDMA, crosslinker), Diethoxyacetophenone (DEOP, Photo-initiator)]. Deionized water were obtained via Milli-Q. Ethanol (AR) and acetone (AR) were supplied by Beijing Chemical Works. All photo images in this work were taken by the photo camera Cannon 60D. All of following ionic liquids were purchased from Lanzhou Yulu Fine Chemical Co. Ltd.. [1-Methyl-3-methylimidazolium bis(trifluoromethylsulfonyl)imide ([C_1MIM][NTf_2], ⩾ 99%), 1-Ethyl-3-methylimidazolium bis(trifluoromethylsulfonyl)imide ([C_2MIM][NTf_2], ⩾ 99%), 1-Propyl-3-methylimidazolium bis(trifluoromethylsulfonyl)imide ([C_3MIM][NTf_2], ⩾ 99%), 1-Ethyl-3-methylimidazolium tetrafluoroborate ([C_2MIM][BF_4], ⩾ 99%), 1-Propyl-3-methylimidazolium tetrafluoroborate ([C_3MIM][BF_4], ⩾ 99%), 1-Butyl-3-methylimidazolium tetrafluoroborate ([C_4MIM][BF_4], ⩾ 99%)， 1-Pentyl-3-methylimidazolium tetrafluoroborate ([C_5MIM][BF_4], ⩾ 99%), 1-Butyl-3-methylimidazolium hexafluorophosphate ([C_4MIM][PF_6], ⩾ 99%), 1-Pentyl-3-methylimidazolium hexafluorophosphate ([C_5MIM][PF_6], ⩾ 99%), 1-Hexyl-3-methylimidazolium hexafluorophosphate ([C_6MIM][PF_6], ⩾ 99%)， 1-Ethyl-3-methylimidazolium trifluoromethanesulfonate ([C_2MIM][OTf], ⩾ 99%), 1-Propyl-3-methylimidazolium trifluoromethanesulfonate ([C_3MIM][OTf], ⩾ 99%), 1-Ethyl-2,3-dimethylimidazolium bis(trifluoromethylsulfonyl)imide ([C_2MMIM][NTf_2], ⩾ 99%), 1-Propyl-2,3-dimethylimidazolium bis(trifluoromethylsulfonyl)imide ([C_3MMIM][NTf_2], ⩾ 99%), 1-Butyl-2,3-dimethylimidazolium bis(trifluoromethylsulfonyl)imide ([C_4MMIM][NTf_2], ⩾ 99%), 1-Propyl-1-methylpyrrolidinium bis(trifluoromethylsulfonyl)imide ([C_3MPY][NTf_2], ⩾ 98%), 1-Butyl-1-methylpyrrolidinium bis(trifluoromethylsulfonyl)imide ([C_4MPY][NTf_2], ⩾ 99%)].

2. Instrumentation

Impedance measurement. The impedance of Cu–ionogel system was investigated using the electrochemical workstation (CHI 604E, Chenhua Instrument, China). The samples were cut into rectangle shape (10 mm in length, 5 mm in width, 2 mm in thickness) and placed between two pieces of copper electrodes.

Surface morphology characterization. The surface morphology of copper electrode were characterized using three-dimensional optical profilometer (Contour GT-X, Bruker). The surface morphology of patterned ionogels were characterized using optical microscope (Eclipse LV100N POL, Nikon).

Nano Computed tomography characterization. The internal structure of phase-separated ionogels were characterized using Nano CT (Nano CT 3DX, Rigaku).

3. Supplementary Figures (1-16)

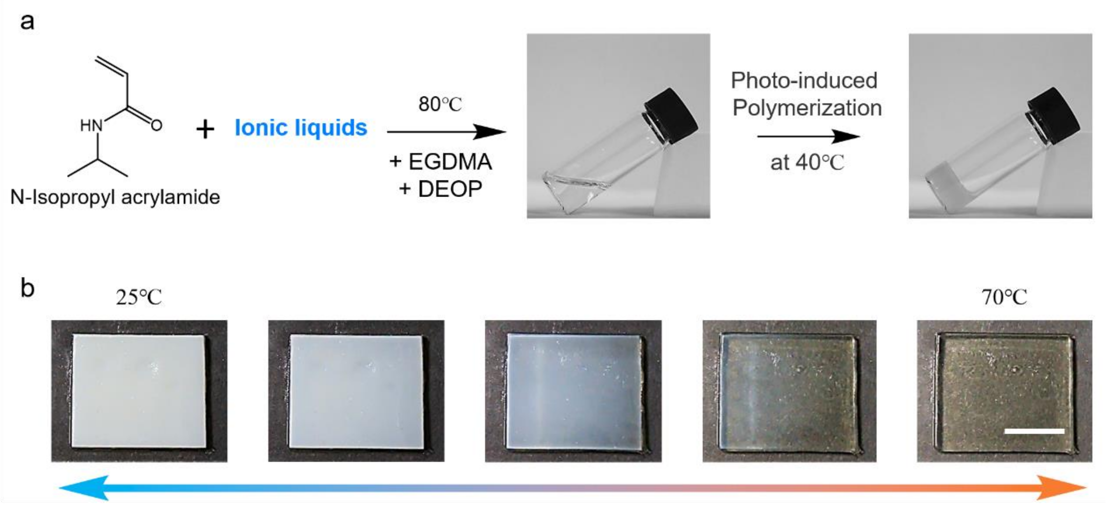

Fig. 1. Preparation of PNIPAm ionogel. (a) Photo-induced polymerization of PNIPAm ionogel. NIPAm monomers were dissolved in ionic liquids ([C_1MIM][NTf_2]) at 80℃. To prevent the crystallization of NIPAm monomers during the preparation of ionogel, the homogenous solution was kept at 40℃ when photo-induced polymerization. The homogeneous solution was curing under UV illumination at 40℃ for 15 min. (b) Photographs show the UCST phase behavior of as-prepared PNIPAm ionogel (20 wt% of NIPAm).

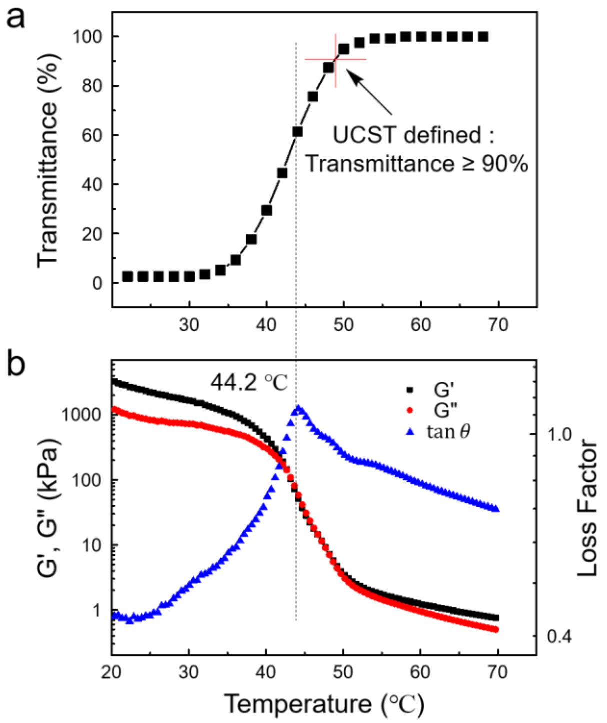

Fig. 2. Thermodynamic behaviors of PNIPAm ionogel. (a) Optical transmittance of ionogel as a function of temperature. (b) Temperature-sweep rheological profiles of PNIPAm ionogel (20 wt% of PNIPAm using [C_1MIM][NTf_2] as solvent). As shown in Fig S2a, the UCST of PNIPAm ionogel is about 49 ℃ (defined as the temperatures at which the transmittance reaches 90%). While the temperature-sweep rheological profiles (Fig S2b) show the ionogel is soften at 44.2 ℃ with an abrupt decrease in the storage modulus (G') and a peak in loss factor (tan δ). These results give an accurate description of the thermoplasticity of PNIPAm ionogel. At room temperature, the glassy state PNIPAm aggregates form a continuous phase in ionogel due to the strong phase separation, resulting in a rigid and opaque plastic. Increasing the temperature to 44.2 ℃, the glassy PNIPAm aggregates destroyed and the mutual miscibility between PNIPAm and ILs increased, resulting in a soft and elastic ionogel. Further increasing temperature above the UCST, ionogel turned into a homogeneous and transparent state.

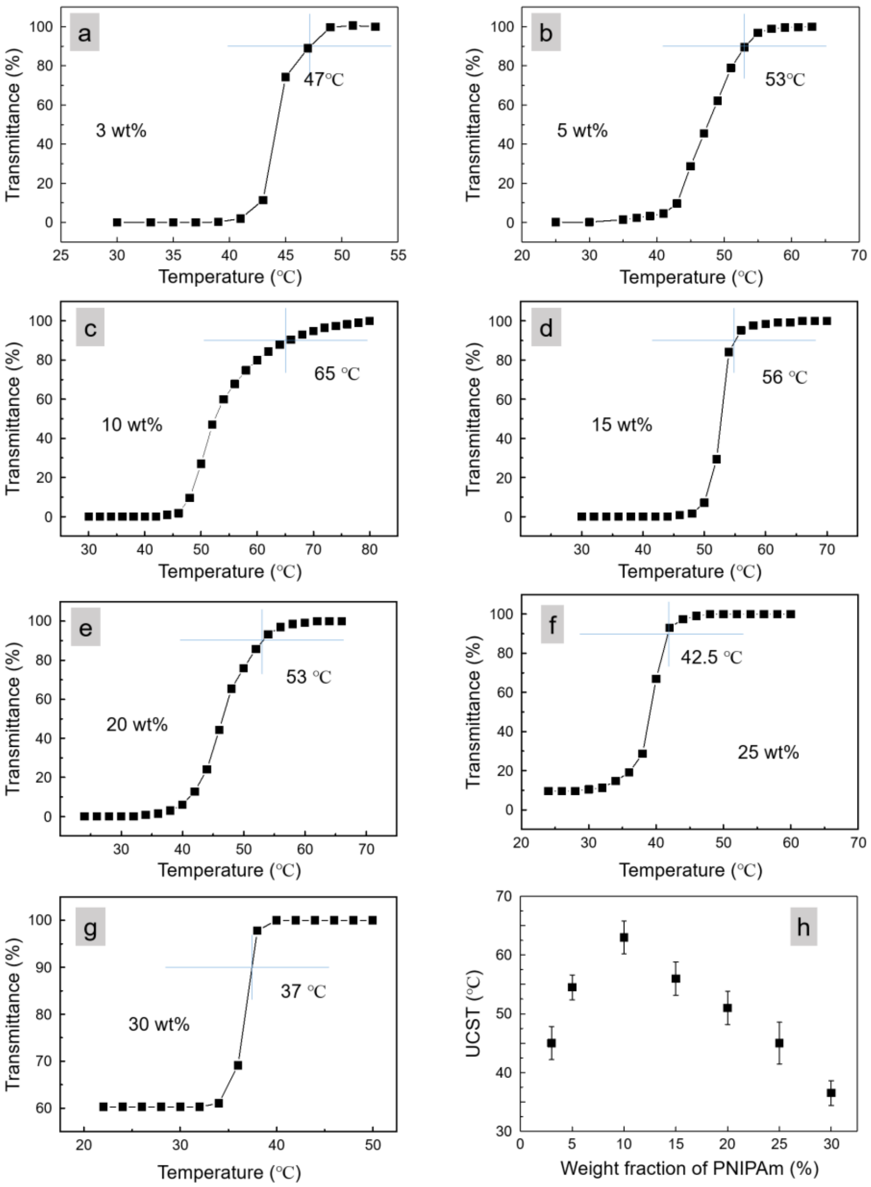

Fig. 3. Temperature dependence of transmittance at 658 nm for ionogels with different PNIPAm content. (a) 3 wt%. (b) 5 wt%. (c) 10 wt%. (d) 15 wt%. (e) 20 wt%. (f) 25 wt%. (g) 30 wt%. (h) UCST of ionogels as a function of the PNIPAm content. For samples with low weight fraction (3 wt% and 5 wt%) of PNIPAm, free standing ionogels can not be obtained. Consequently, binary PNIPAm/ILs mixture were used to characterize their UCST. Ionogels with high PNIPAm content (more than 10 wt%) were cut into rectangle shape (20 mm ×8 mm ×1.5 mm) and placed on the inner wall of cuvette. We define the CP (i.e. UCST) values as the temperatures at which the transmittance reaches 90%. All error bars represent the mean ± standard deviations (n ⩾ 3 independent experiments).

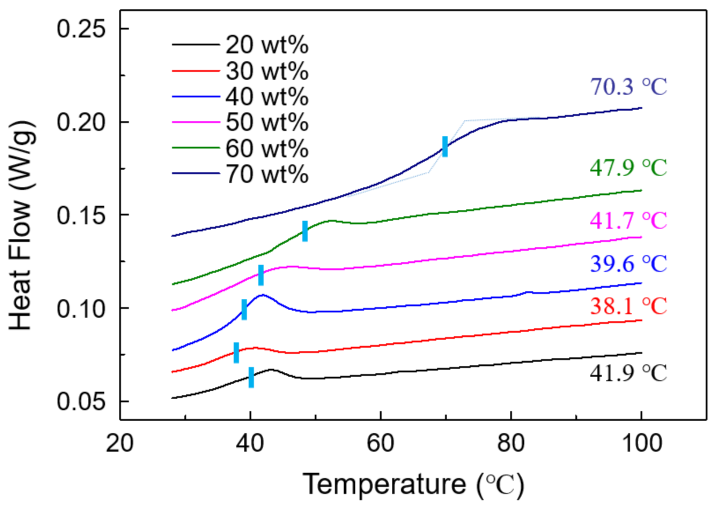

Fig. 4. DSC profiles of ionogels with different PNIPAm content ([C_1MIM][NTf_2]). The glass transition temperature of PNIPAm ionogels were indicated on each DSC curves.

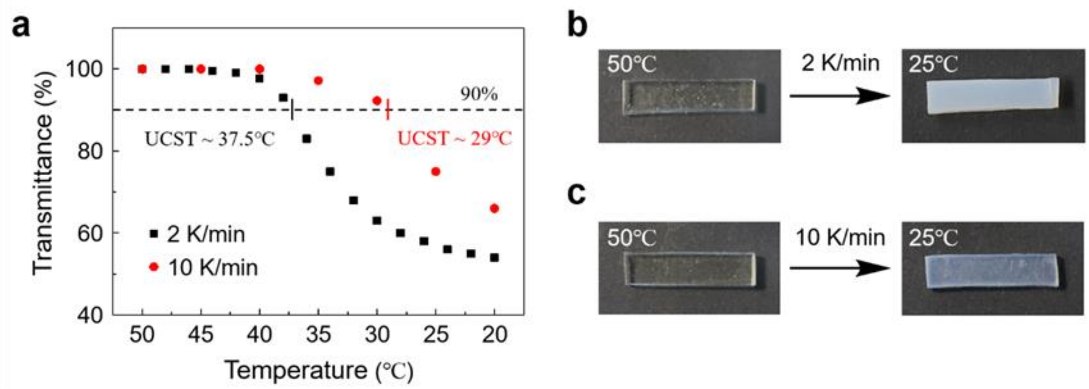

Fig. 5. Temperature dependence of transmittance at 658 nm for PNIPAm ionogels under different cooling rate. (a) We define the cloud point (i.e. UCST) values as the temperatures at which the transmittance below 90%. (b) and (c) Photographs showing ionogels treated with different cooling rates.

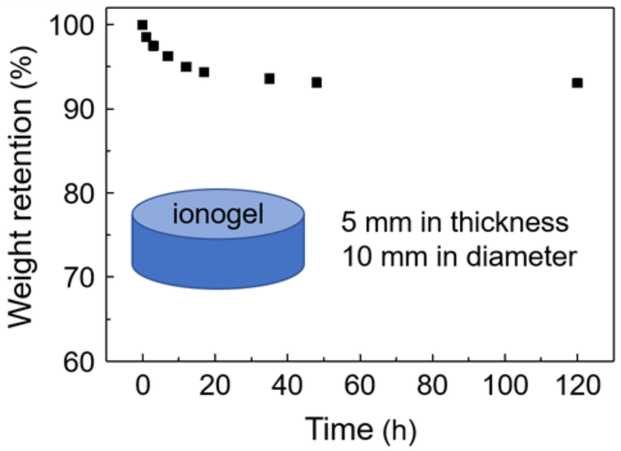

Fig. 6. Weight retention of phase-separated ionogel as a function of time (25 wt% PNIPAm/[C_1MIM][NTf_2] ionogel). Taking 25 wt% PNIPAm/[C1MIM][NTf2] ionogel (UCST ~ 45℃) as an example, a time dependent mass variation of phase-separated ionogel at room temperature was recorded. For current sample, only less than 7 wt% solvent was expelled from the phase-separated ionogel (i.e. T<UCST) when held at room temperature for 4 days. Consequently, the solvent trapping degree (D_{st}) can be define as follow:

$$\mathrm{D}_{st} = 1 - \frac{\text{Solvent loss}}{\text{Total solvent content}} = 1 - \frac{7\%}{75\%} = 90.7\%$$

It is worth noting that though the frozen of polymer-dense phase can keep the volume unchanged of ionogel in macroscale, the solvent at the superficial zone of ionogel will be inevitably expelled from the ionogel. Therefore, it can be concluded that ionogel with smaller specific surface area will exhibit more excellent solvent trapping capability (when held at phase-separated state).

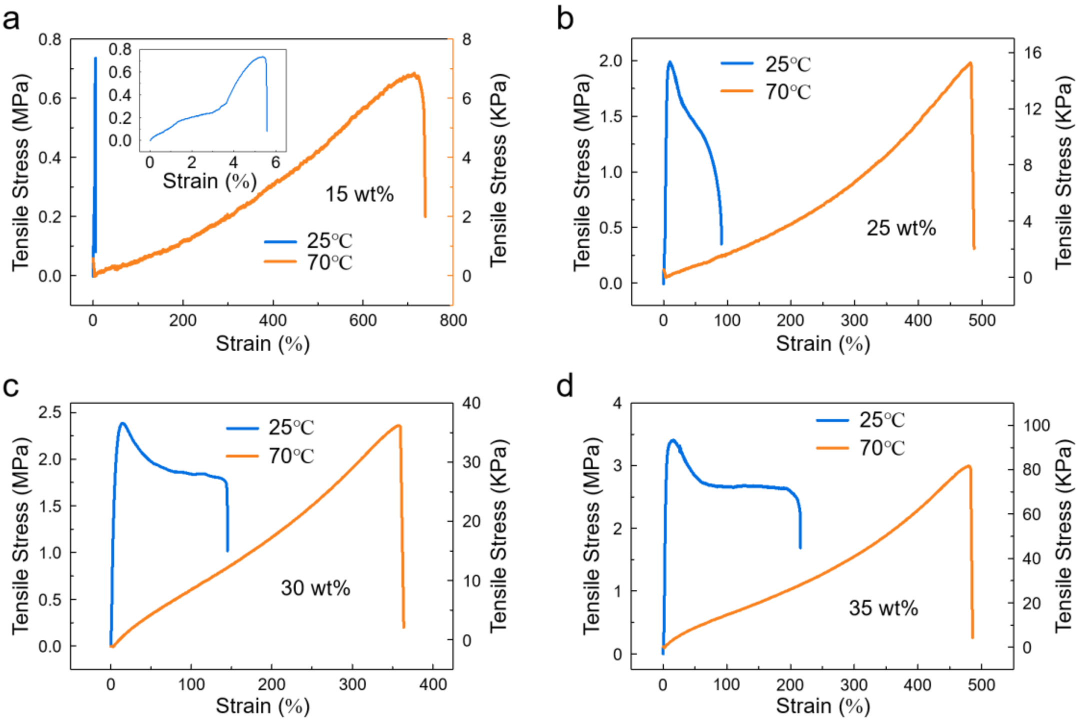

Fig. 7. Tensile stress-strain curves of PNIPAm ionogels with different PNIPAm content ($[C_1MIM][NTf_2]$). (a) 15 wt%, (b) 25 wt%, (c) 30 wt%, (d) 35 wt%.

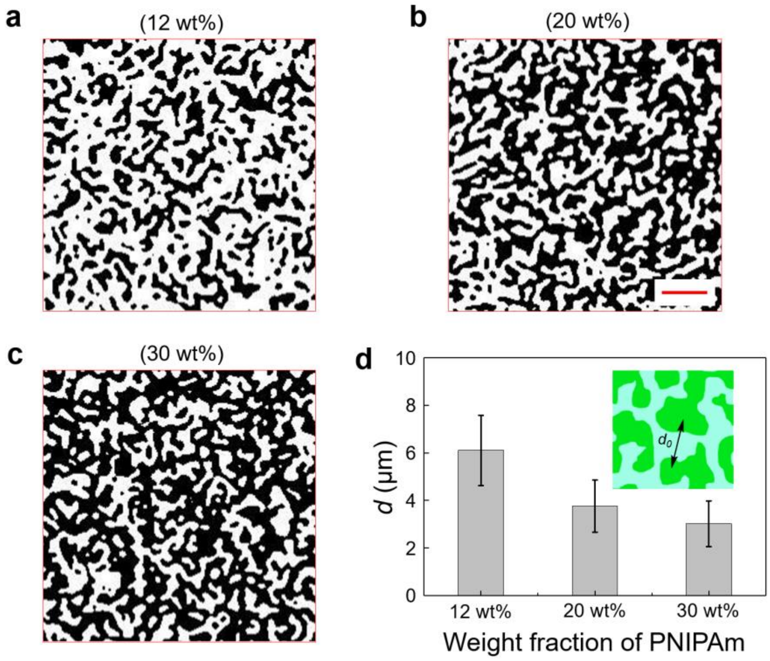

Fig. 8. Extracted images of 2D filling ratio from Nano CT scanning base on the difference in density (under a selected threshold). (a)-(c) 2D Nano CT images of PNIPAm ionogels with different polymer content. The white and black areas represent the heavy (solvent-rich region) and light (polymer-rich region) element, respectively. (d) Average value of d_0 varies with the polymer content. d_0 is the spacing between adjacent polymer-rich (or solvent-rich) regions. These results qualitatively indicate the volume increase of vitrified-polymer-dense phase as increasing the polymer content from 12 wt% to 30 wt% (scale bar 20 μm). All error bars represent the mean ± standard deviations (n ⩾ 3 independent experiments).

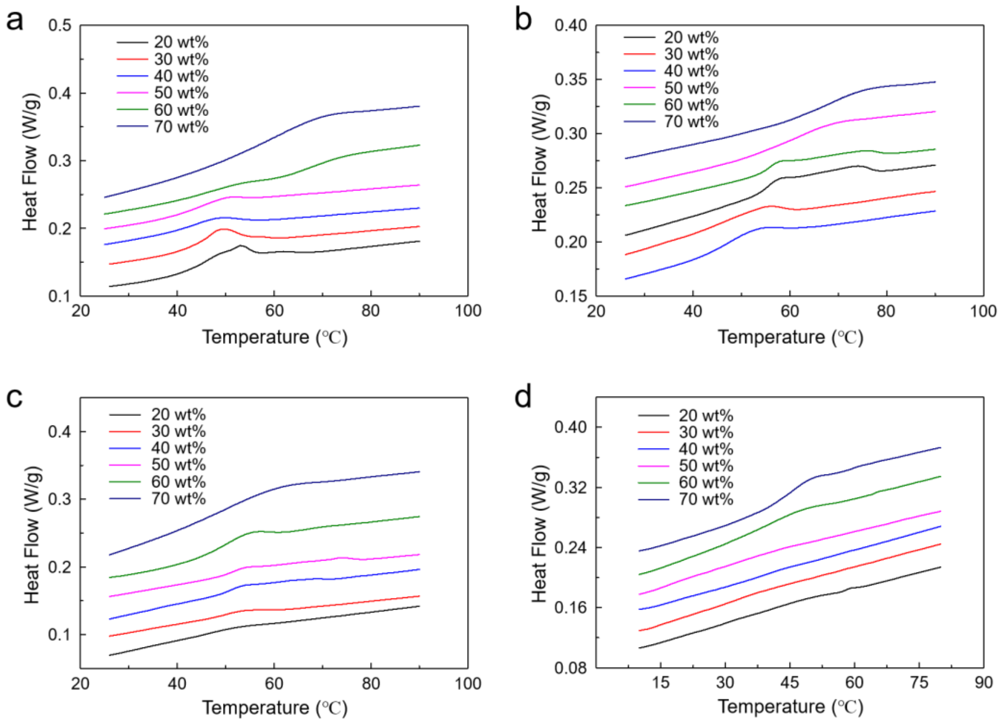

Fig. 9. DSC profiles of PNIPAm ionogels. (a) DSC results of PNIPAm ionogels using [C_3MPY][NTf_2] as solvent. (b) DSC results of PNIPAm ionogels using [C_3MMIM][NTf_2] as solvent. (c) DSC results of PNIPAm ionogels using [C_5MIM][PF_6] as solvent. (d) DSC results of PNIPAm ionogels using [C_5MIM][BF_4] as solvent.

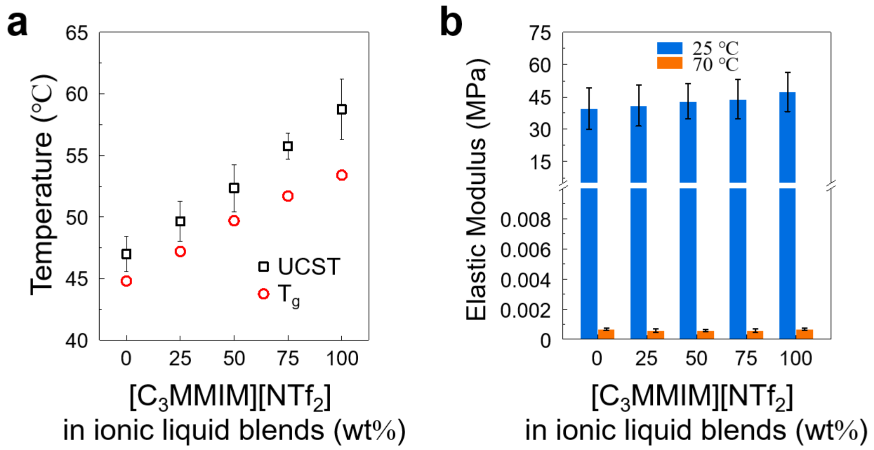

Fig. 10. T_g, UCST and mechanical performance of PNIPAm ionogel. (a), Tuning the UCST and T_g of ionogels by varying the mixing ratio of cations in ionic liquids blends ($[C_3MMIM][NTf_2]/[C_3MPY][NTf_2]$). (b), Thermoresponsive stiffness-changing properties of the ionogels in **a** (PNIPAm content: 20 wt%). All error bars represent the mean ± standard deviations (n $\geqslant$ 3 independent experiments).

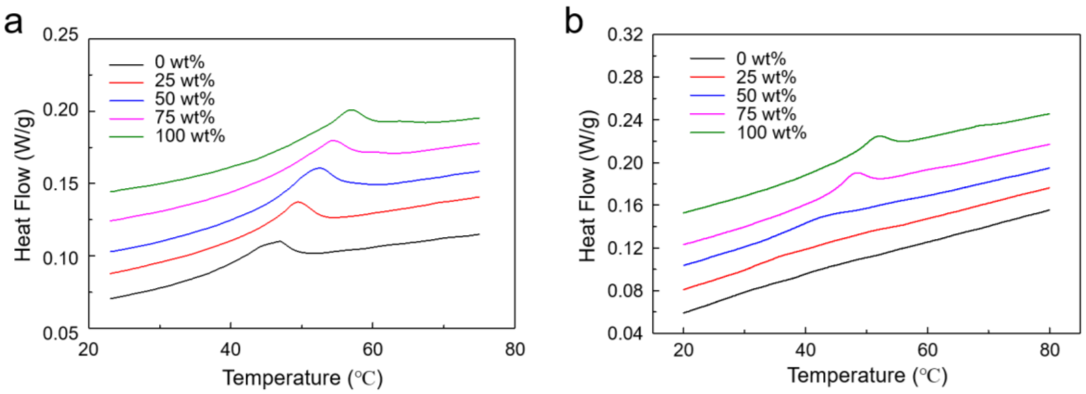

Fig. 11. DSC profiles of PNIPAm ionogels. (a) DSC results of PNIPAm ionogels using ionic liquid blends ([C_3MMIM][NTf_2]/[C_3MPY][NTf_2]) as solvent (weight fraction of [C_3MMIM][NTf_2] in their blends). By varying the combination of the cation in their blends, the T_g of PNIPAm ionogels can be tuned to exhibit a linear variation from 44.8℃ to 53.4℃. (b) DSC results of PNIPAm ionogels using ionic liquid blends ([C_5MIM][PF_6]/[C_5MIM][BF_4]) as solvent (weight fraction of [C_5MIM][PF_6] in their blends). By varying the combination of the anion in their blends, the T_g of PNIPAm ionogels can be tuned from RT to 50.5℃.

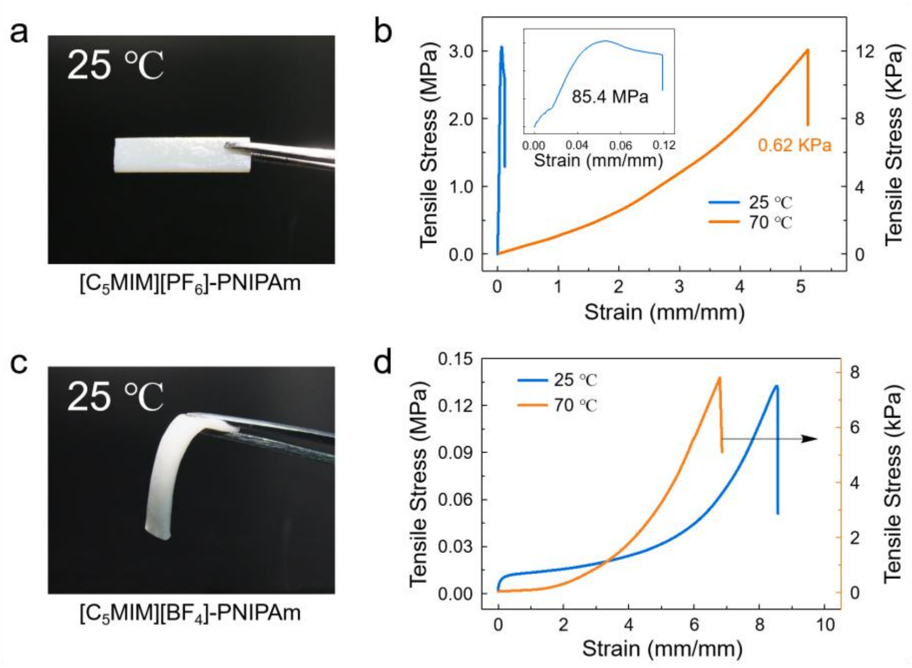

Fig. 12. Mechanical characterization of PNIPAm ionogels. (a) Photograph of phase-separated [C_5MIM][PF_6]-PNIPAm ionogels (20 wt% PNIPAm content, UCST ~ 66.3°C). (b) Stress-strain curves of [C_5MIM][PF_6]-PNIPAm ionogels at RT and 70°C. (c) Photograph of phase-separated [C_5MIM][BF_4]-PNIPAm ionogels (20 wt% PNIPAm content, UCST ~ 58.3°C). (d) Stress-strain curves of [C_5MIM][BF_4]-PNIPAm ionogels at RT and 70°C.

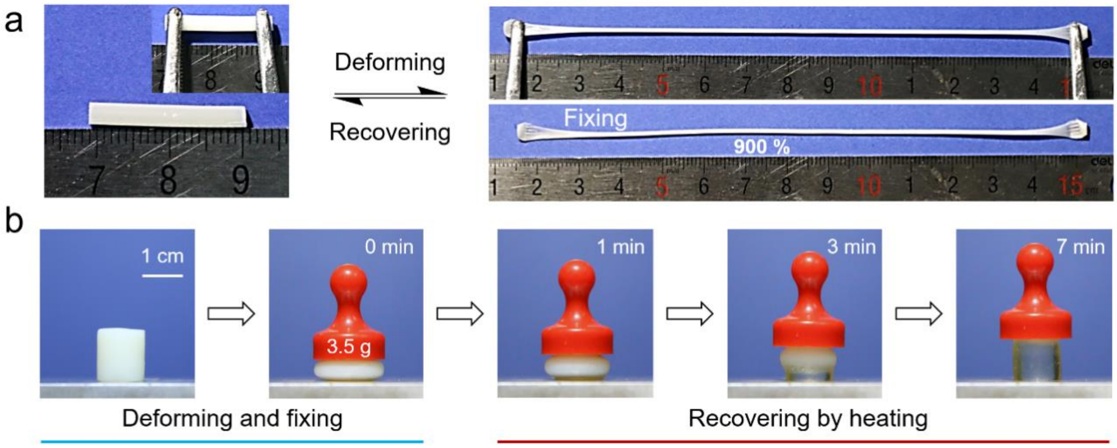

Fig. 13. Applications of PNIPAm ionogels. (a) Large-strain shape memory (900%). (b) Mechanical energy storage. (Ionogels composed of 20 wt% PNIPAm and $[C_1MIM][NTf_2]$).

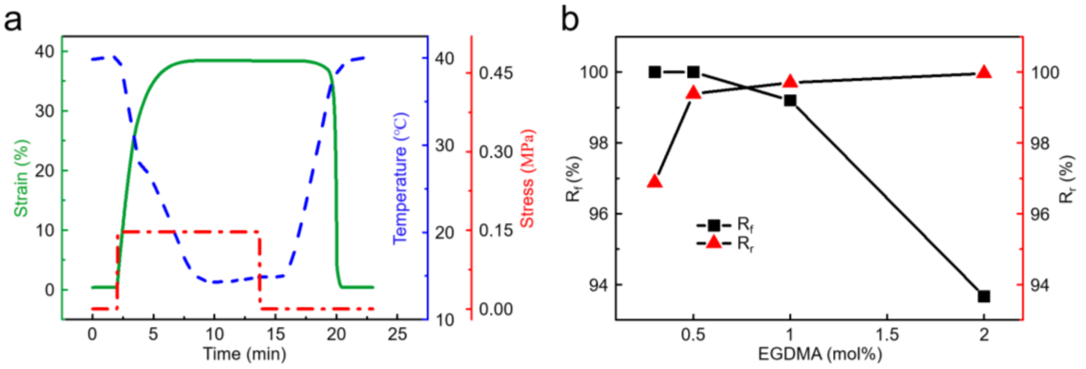

Fig. 14. The shape memory behavior of PNIPAm ionogel is characterized by quantitative thermal mechanical cycles using dynamic thermomechanical analysis. (a) Quantitative thermal mechanical cycles for shape memory properties of PNIPAm ionogel. (b), R_f and R_r results from the tensile test with respect to different crosslinking density of the PNIPAm ionogel. (Ionogel composed of 20 wt% PNIPAm and $[C_1MIM][NTf_2]$).

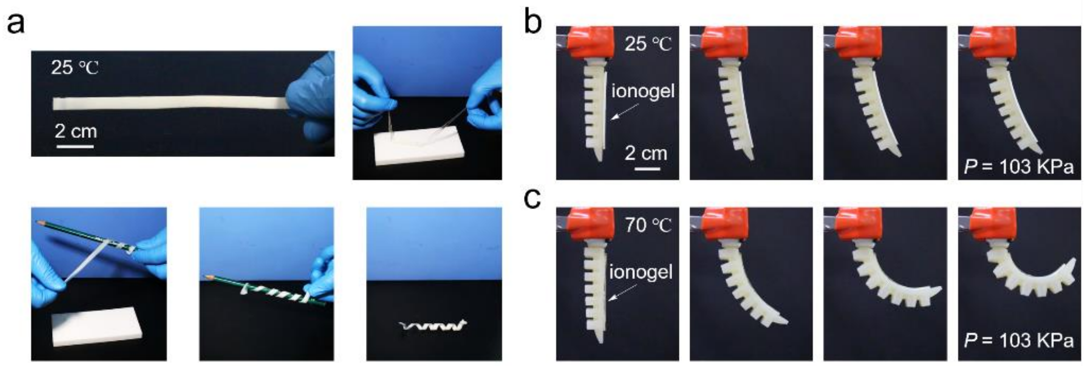

Fig. 15. Applications of PNIPAm ionogels. (a) Demonstration of ionogel that can be used as an intelligent gripper. In soft state, the ionogel can be deformed and wrapped on the target object. Cooling at RT, the coil shape ionogel is fixed and hard enough to bear the weight of the target object. (b) and (c) By attaching a thin ionogel layer on the one side of the pneumatic device, a smart pneumatic-thermal hybrid actuation device is realized. The different bending angle of the soft gripper at constant pneumatic pressure (P = 103 kPa) is originated from the thermo-induced stiffness-changing of the PNIPAm ionogel. (Ionogel composed of 20 wt% PNIPAm and [C_1MIM][NTf_2]).

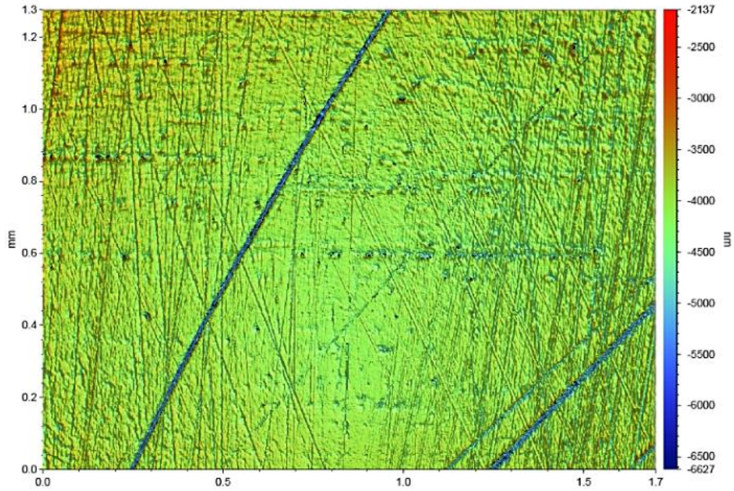

Analytical Results

Label	Value	Units
Average	-4019.374	nm
Data Points	1225127.04	
Percent Data Points	99.71	%
Ra	4019.374	nm
Rp	-2137.052	nm
Rq	4026.013	nm
Rt	4489.993	nm
Rv	-6627.044	nm

Fig. 16. Surface morphology of copper electrode used in Figure 4 in the main text. The surface morphology of copper electrode were characterized using three-dimensional optical profilometer (Contour GT-X, Bruker). As shown in the analytical results (right table), the surface roughness $R_a \sim 4$ µm.

4. Supplementary Tables (Table 1-Table 3)

	E_{hard} (kPa)	E_{soft} (kPa)	Max. stiffness change (E_{hard}/E_{soft})	Solvent content (wt%)	Reference
**Bottlebrush elastomers**	230 x 10^3	6	38333	0	[1]
**Shape memory polymers**	950 x 10^3	2.0 x 10^3	475	0	[2]
	102 x 10^3	135 x 10^2	75	0	[3]
**Crystalline gels**	474 x 10^3	18	26346	36	[4]
	385 x 10^3	15	25666	8.3	[5]
	601 x 10^3	4.5	133555	15	[6]
**Phase change materials microcomposites**	887.9 x 10^3	1250	> 700	30	[7]
	3100	100	31	50	[8]
	40 x 10^3	1.5 x 10^3	25	38	[9]
	950 x 10^3	30	31666	30	[10]
	860 x 10^3	100	8600	39	[11]
**Polyelectrolyte gels**	5400	1	5400	60	[12]
	4400	8	550	53.2 ~ 66.9	[13]
	56.3 x 10^3	1140	50	51.7 ~ 66.4	[13]
**Organohydrogels**	570	53	10.7	69	[14]
	1200	100	12	55	[15]
**Thermal hardening hydrogels**	119 x 10^3	70	1800	74	[16]
**Inorganic stimuli-responsive hydrogels**	600	26	23	86	[17]
**Phase-separated hydrogels**	30	5	6	82	[18]
	40	4	10	80	[19]
**Agar/PAM DN gels**	123	34	3.6	77	[20]

Table 1. Comparison of the stiffness-changing property of polymer content materials and references for Figure 2F in the main text.

	UCST (℃)	$E_{25℃}$ (MPa)	$E_{70℃}$ (kPa)	$E_{25℃}/E_{70℃}$	$\sigma_{25℃}$ (MPa)	$\sigma_{70℃}$ (kPa)	$\sigma_{25℃}/\sigma_{70℃}$	$WoE_{25℃}$ (MJ/m^3)	$WoE_{70℃}$ (MJ/m^3)	$WoE_{25℃}/WoE_{70℃}$
PNIPAm/ $[C_1MIM][NTf_2]$	50.1	62.5	0.66	94697	1.02	7	146	1.31	0.013	**100.7**
PNIPAm/ $[C_5MIM][BF_4]$	58.3	2.18×10^{-3}	0.53	4.11	0.132	8.3	15.8	0.33	0.015	22
PNIPAm/ $[C_5MIM][PF_6]$	68.3	85.4	0.62	**137741**	2.56	11.7	**218.8**	0.27	0.023	11.87

Table 2. Mechanical properties of three representative samples at 25 ℃ and 70 ℃ are summarized. $E_{25℃}$ and $E_{70℃}$: Young's moduli of at 25 ℃ and 70 ℃, respectively; $\sigma_{25℃}$ and $\sigma_{70℃}$: fracture stresses at 25 ℃ and 70 ℃, respectively. $WoE_{25℃}$ and $WoE_{70℃}$: work of extension (*WoE*) at 25 ℃ and 70 ℃, respectively (*WoE* was defined as the area under the stress-strain curve).

Materials	Stiffness-changing ratio: R (E_{hard}/E_{soft})	Responsive temperature: T_{soft} [°C]	Temperature sensitivity: R/ΔT [1/°C]	Ionic conductivity
**Our ionogels**	**4403 ~ 137741 (tunable)**	**37 ~ 70 (tunable)**	**367 ~ 2504 (tunable)**	**Yes**
Phase separated hydrogel	~1800	70	40	No
Shape memory polymer	~ 475	130	6.3	No
Phase change polymer composite	~ 31	62	0.8	No
Bottlebrush elastomers	~3300	37	275	No

Table 3. Thermomechanical performance of polymer-based stiffness-changing materials. The resulting ionogel shows unprecedented properties (stiffness-changing ratio and temperature sensitivity) that surpass all reported polymer-based stiffness-changing materials ($\Delta T = T_{soft} - T_{hard}$: Temperature difference between two stable states).

5. References

1. D. X. Zhang et al., Tissue-Adaptive Materials with Independently Regulated Modulus and Transition Temperature. Advanced Materials 32, 2005314 (2020).
2. Xie, T. Tunable polymer multi-shape memory effect. Nature 464, 267-270 (2010).
3. A. Balasubramaniam, M. Standish, C. J. Bettinger, Microfluidic Thermally Activated Materials for Rapid Control of Macroscopic Compliance. Advanced Functional Materials 24, 4860-4866 (2014).
4. Wei, J. et al. Dissolution–Crystallization Transition within a Polymer Hydrogel for a Processable Ultratough Electrolyte. Advanced Materials 31 (2019).
5. Yang, F. K., Cholewinski, A., Yu, L., Rivers, G. & Zhao, B. A hybrid material that reversibly switches between two stable solid states. Nature Materials 18, 874 (2019).
6. Zhao, X. et al. Phase change mediated mechanically transformative dynamic gel for intelligent control of versatile devices. Materials Horizons 8, 1230-1241 (2021).
7. A. Tonazzini et al., Variable Stiffness Fiber with Self-Healing Capability. Advanced Materials 28, 10142-10148 (2016).
8. I. V. Meerbeek et al., Morphing Metal and Elastomer Bicontinuous Foams for Reversible Stiffness, Shape Memory, and Self-Healing Soft Machines. Advanced Materials 28, 2801-2806 (2016).
9. B. E. Schubert, D. Floreano, Variable stiffness material based on rigid low-melting-point-alloy microstructures embedded in soft poly(dimethylsiloxane) (PDMS). Rsc Advances 3, 24671-24679 (2013).
10. S. H. Byun, J. Y. Sim, Z. Zhou, J. Lee, J. W. Jeong, Mechanically transformative electronics, sensors, and implantable devices. Science Advances 5, eaay0418 (2019).
11. W. Shan, T. Lu, C. Majidi, Soft-matter composites with electrically tunable elastic rigidity. Smart Material Structures 22, 085005 (2013).
12. Luo, F. et al. Oppositely Charged Polyelectrolytes Form Tough, Self-Healing, and Rebuildable Hydrogels. Advanced Materials 27, 2722-2727 (2015).
13. F. Luo et al., Tough polyion-complex hydrogels from soft to stiff controlled by monomer structure. Polymer, 116, 487-497 (2017).
14. Z. Zhao, K. Zhang, Y. Liu, J. Zhou, M. Liu, Highly Stretchable, Shape Memory Organohydrogels Using Phase-Transition Microinclusions. Advanced Materials 29, 1701695 (2017).
15. S. Zhuo, Z. Zhao, Z. Xie, Y. Hao, M. Liu, Complex multiphase organohydrogels with programmable mechanics toward adaptive soft-matter machines. Science Advances 6, eaax1464 (2020).

16. T. Nonoyama, W. L. Yong, K. Ota, K. Fujioka, P. G. Jian, Instant Thermal Switching from Soft Hydrogel to Rigid Plastics Inspired by Thermophile Proteins. Advanced Materials 32, 1905878 (2020).
17. K. Sano, N. Igarashi, Y. Ebina, T. Sasaki, Y. Ishida, A mechanically adaptive hydrogel with a reconfigurable network consisting entirely of inorganic nanosheets and water. Nature Communications 11, 6026 (2020).
18. L. W. Xia et al., Nano-structured smart hydrogels with rapid response and high elasticity. Nature Communications 4, 2226 (2013).
19. G. Hui, N. Sanson, D. Hourdet, A. Marcellan, Thermoresponsive Toughening with Crack Bifurcation in Phase-Separated Hydrogels under Isochoric Conditions. Advanced Materials 28, 5857-5864 (2016).
20. C. Qiang, L. Zhu, C. Zhao, Q. Wang, Z. Jie, A robust, one-pot synthesis of highly mechanical and recoverable double network hydrogels using thermoreversible sol-gel polysaccharide. Advanced Materials 25, 4171-4176 (2013).
